# Dumping military waste into Lake Superior: the historic legacies of secrecy, censorship, and uncertainty

**DOI:** 10.1007/s12685-023-00329-y

**Published:** 2023-05-30

**Authors:** Elodie Charrière, Nancy Langston

**Affiliations:** 1 Department of Social Sciences, Michigan Technological University, Houghton, MI USA; 2 Environmental Governance and Territorial Development Hub/Institute, Institute for Environmental Sciences, University of Geneva, Geneva, Switzerland

**Keywords:** Underwater military waste, Lake Superior, Water pollution, Environmental history

## Abstract

In recent years, the issue of military waste disposal in oceans and seas has gained significant attention; however, the impact of such waste in freshwater deposits has been understudied. The Laurentian Great Lakes of North America contain 20% of the world’s fresh surface water and are particularly vulnerable to environmental stressors such as climate change, invasive species, and toxic chemicals, making the examination of military waste management in these waters crucial. This interdisciplinary study aims to investigate the legacy of two military waste disposal sites in Lake Superior, referred to as Site A (containing barrels) and Site B (containing bullets). Both are located within the ceded territories of the Ojibwe. Despite being in close proximity, these sites have had vastly different outcomes in terms of public concern, state and federal regulatory actions, and tribal restoration efforts. Based on this observation, this study aims to answer the following questions: How did these differences develop? How did military secrecy and the loss of memory influence the management of underwater military waste at each site? How do uncertainties and rumors continue to influence citizen concern and agency management of military waste? We argue for the importance of investigating the environmental legacies of underwater military waste in order to protect inland freshwater resources worldwide.

## Introduction

Until the 1970s, dumping military waste in aquatic environments was a common method of disposal. Historians, scientists, and engineers have conducted historical research and explored the historic context and environmental concerns related to marine dumping and its regulation in the 1970s (Arison 2013; Carton and Jagusiewicz 2009; Hamblin 2008; Jenks 2007; Mitchell 2020; Müller 2016; Souchen 2017, 2020, 2021a, 2021b). However, fewer historians have examined military waste disposal within freshwater lakes, apart from military dumping in Swiss lakes (Charrière 2019).

**Fig. 1:**
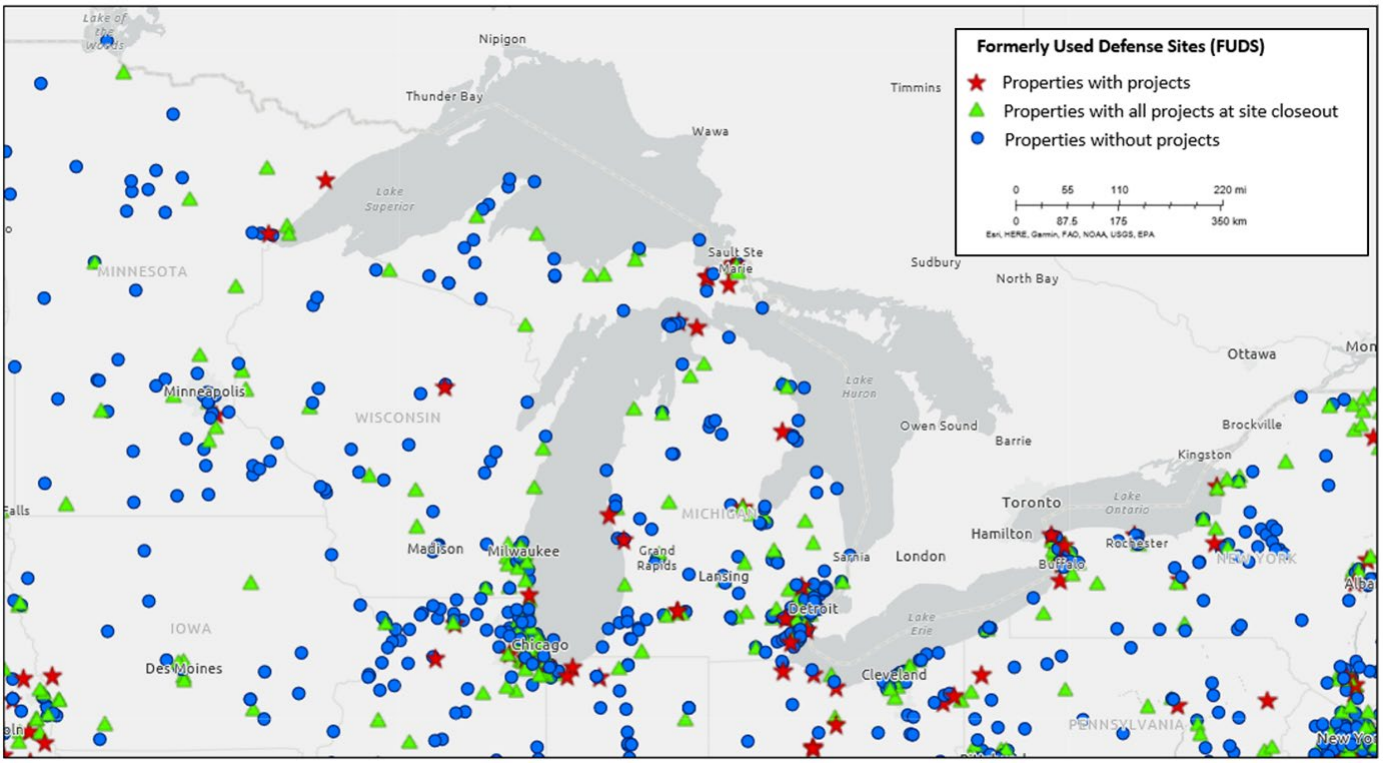
Formerly Used Defense Sites on the U.S. Side of the Great Lakes Region. Credit: U.S. Army Corps of Engineers

The Laurentian Great Lakes of North America hold 20% of the world’s fresh surface water. Their waters seemed like a convenient sink for industrial wastes in the nineteenth and early twentieth century (Benidickson 2007; Colten 1986; Hites 2006; Kehoe 1992; Tarr 1996). Regional pollution from industrial and urban waste sparked grassroots concerns and environmental regulation in the post-World War II era (Ashworth 1987; Bogue 2000; Kehoe 1997; Langston 2017). Military pollution in the region, however, received less attention (Albright 2012; Nathan and Bronstein 1998). In part, the absence of regional military conflicts in the twentieth century helps explain this lack of concern. Yet both Canada and the United States 1 developed extensive military infrastructure in the Great Lakes region, including munitions plants, training sites, depots, and air bases (see Fig. 1). All of these sites had to dispose of their wastes (including conventional and chemical munitions) and dumping in the local waters sometimes seemed to offer a cheaper and safer alternative to incineration or burial.

In the 1990s, a former U.S. Army Corps of Engineers (Corps) supervisor reported to a Minnesota Pollution Control Agency (MPCA) employee that there were at least 16 disposal sites within Lake Superior where the Army had dumped everything imaginable (*St. Paul Pioneer Press*, November 28, 1993). Where are they? What are they made of? Which substances are lying at the bottom of Lake Superior? What environmental impacts can result from such disposal sites? Thirty years later, most of those questions are still unanswered.

**Fig. 2:**
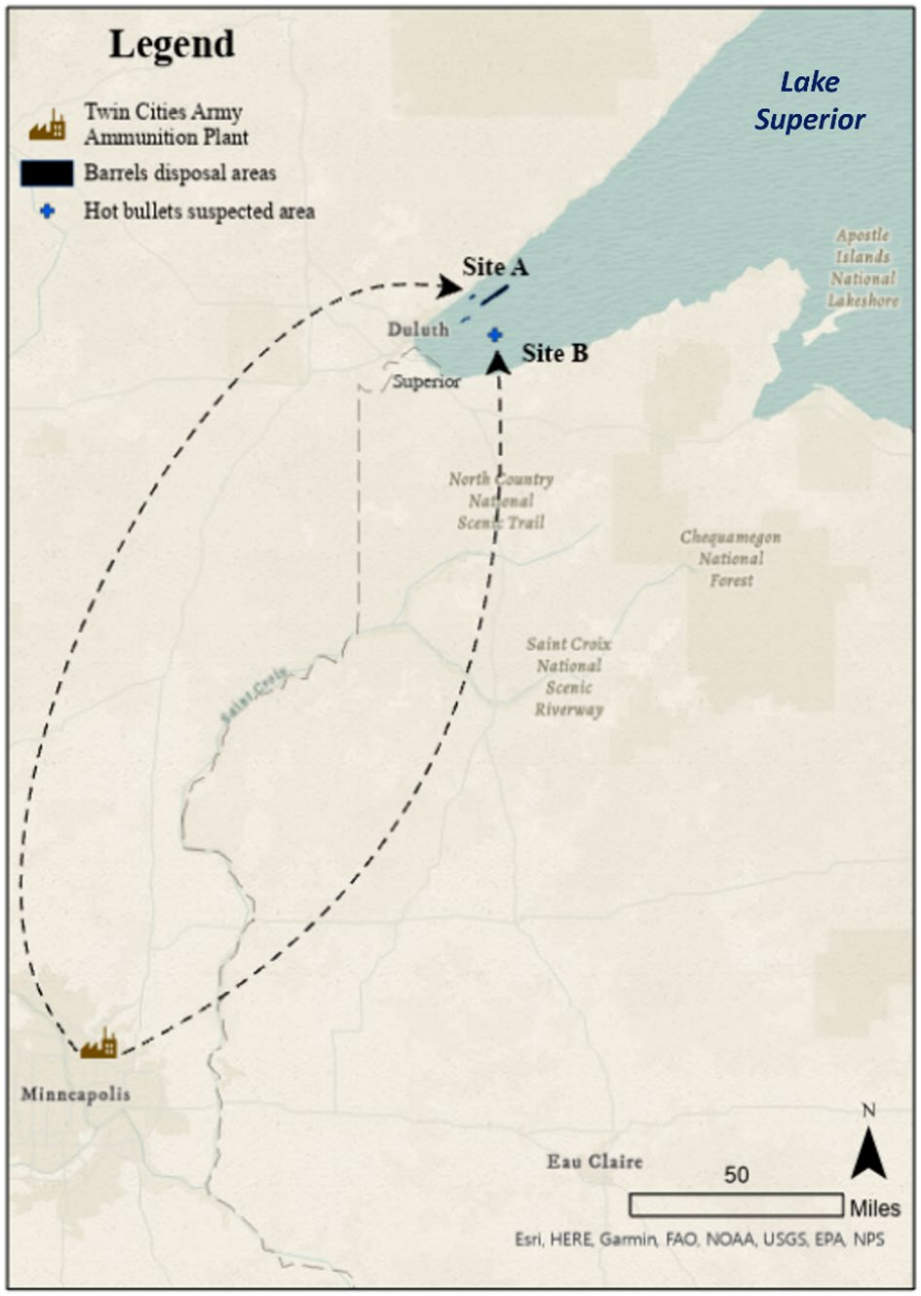
Two Sites in Lake Superior with Underwater Military Wastes. Site A: Barrels; Site B: Bullets

This article examines two military waste dumping sites within Lake Superior, the largest freshwater lake by surface area in the world (see Fig. 2). The Great Lakes are international waters, and the International Joint Commission exercises some transnational management oversight over their water quality. The sites discussed in this article lie within the ceded territories of the Ojibwe (1842 Treaty), and tribal nations now play key roles in ensuring water quality restoration within those ceded territories.

Both sites we discuss contain waste from the Twin Cities Army Ammunition Plant (TCAAP), near Minneapolis, Minnesota (see Fig. 2). TCAAP is notorious for becoming Minnesota’s largest Superfund Site. At Site A, approximately 1437 barrels of military waste were dumped into Lake Superior between October 1959 and September 1962, representing over 830,000 pounds of classified grenade scrap. Rumors of radioactivity sparked decades of public concern and millions of dollars poured into inconclusive cleanups that satisfied few citizens. In contrast, at Site B, over 1 million pounds of bullets filled with highly explosive incendiary powder were dumped in boxes in Fall 1945, but this site has attracted little concern and even less cleanup funding.

The two sites are extremely close together in Lake Superior, and the materials come from the military facility. Yet the two sites have had quite different fates, with respect to public concern, state and federal regulatory actions, and tribal restoration efforts. While this article focuses mainly on Site A (Fig. 2), we use Site B (Fig. 2) to contrast the historical legacies associated with military waste management practice. We ask: How did these differences develop? How did military secrecy and the loss of memory influence the management of underwater military waste at each site? How do uncertainties and rumors continue to influence citizen concern and agency management of military waste?

This paper argues that several factors can explain the heterogeneous management applied to Sites A and B, despite their similarities (dumping of military waste from the same site) and their proximity within Lake Superior. First, we argue that the different historic contexts in which these dumping occurred shaped their different outcomes. The dumps at Site B were conducted openly in September 1945, when the military enjoyed widespread positive support from the population and was widely celebrated for its role in achieving victory. In contrast, the dumping of barrels at Site A occurred during the Cold War period in a context of suspicion, and the military conducted the operations in secrecy. We argue that this secrecy promoted rumors about the contents of the barrels and undermined trust in military discourse. By contrasting the management and controversies regarding these two military dump sites within Lake Superior, we show that secrecy, citizen activism, media responses, and tribal concerns influenced the military’s treatment of wastes dumped into the world’s largest freshwater lake.

## Methodology

The methods of this study are interdisciplinary, combining archival analysis, scientific literature review, and interviews. We reviewed federal and state agency, tribal authority, and environmental NGO reports regarding these sites. We used Freedom of Information Act (FOIA) requests to access documents from the Minnesota Pollution Control Agency, the U.S. Environmental Protection Agency (EPA) Region 5, and the U.S. Army Corps of Engineers, Omaha District. We also used the portal developed by the U.S. Army Corps of Engineers, Engineer Research and Development Center—Information Technology Laboratory to explore concerns regarding formerly used defense site (FUDS) data.^2^ We visited archives at the Duluth Public Library (DPL), the Minnesota Historical Society, the Kathryn A. Martin Library and Archives (KAML&A), the Michigan Technological University Archives (MTUA), and the National Archives (NARA) at Chicago. We analyzed historic newspaper articles and conducted interviews^3^ with Linda Nguyen (environmental director at Red Cliff Band of Lake Superior Chippewa), Dan Rau (former captain, former researcher and lecturer at the University of Wisconsin Superior, and environmental advocate in Save Lake Superior Association) and Marilyn Burton (a citizen advocate living in Sault Ste Marie, MI).

## Lake superior characterization

Lake Superior is the largest and most northwesterly of the five Great Lakes in North America and is the world’s largest (by surface area) freshwater lake. It is a transnational lake, governed by both the United States and Canada, and by Indigenous communities that continue exercise their sovereign treaty rights in the basin. The lake covers an area of 31,700 square miles, with a draining basin of 49,300 square miles. Its average depth is 483 feet, with a maximum depth of 1332 feet. The average temperatures have been extremely cold (39 °F or 4 °C average subsurface temperatures). As a result, Lake Superior is ultra-oligotrophic, meaning that it is low in productivity and high in dissolved oxygen. The water retention time of the lake is estimated to be 191 years, so pollutants in the lake are retained for nearly two centuries (Langston 2017).

Although it is the least industrialized of all the Great Lakes, environmental stressors are degrading its ecosystem. These stressors include chemical contamination (e.g., mercury, PCBs, toxaphene), shoreline development, invasive species (e.g., sea lamprey, round goby, brown trout), habitat loss, wetland loss and degradation, and forest fragmentation. The lake is also one of the fastest warming large lakes in the world. Shrinking ice cover, intensified precipitation, and higher water temperatures are major concerns for sensitive species and winter tourism (Langston 2017).

## Twin cities army ammunition plant (TCAAP)

Small arms ammunition is defined as projectiles with a diameter measuring no more than six-tenths of an inch, equivalent to 0.60 caliber or less. In 1941, before the United States entered World War II, military production of small arms ammunition was extremely limited. Only one plant in the U.S. was producing them. In May 1941, the War Department declared the construction and equipment of small arms munition a defense priority. Three months later, construction of the Twin Cities Army Ammunition Plant (TCAAP) as a government-owned, contractor-operator^4^ facility started in Minnesota (see Fig. 2). The Federal Cartridge Company was the plant’s civilian contractor during the initial years of construction and operation, assuming responsibility for operating the plant and managing waste disposal. On February 2, 1942, the production of small arms ammunition began, continuing for over 43 months until September 28, 1945. Located in New Brighton and Arden Hills, the plant was approximately 8 miles from the Mississippi river and 13 miles north of the Twin Cities, the largest cities in Minnesota.

After World War II ended, the plant returned to standby status with Army oversight. In subsequent wars, the plant returned to military production under the supervision of Federal Cartridge Company. In August 1950, the facility began manufacturing small arms and artillery ammunition for the Korean War. In 1957, the plant closed again after the war ended, only to reopen in 1965 during the Vietnam War. The TCAAP was put on standby in 1976 and closed officially in 1993. That same year, the Army announced plans to release portions of TCAAP as excess federal property to allow for civilian redevelopment.

From 1942 to 1972, TCAAP produced ammunition intermittently, and production levels varied over time. Nonetheless, the quantity of small arms ammunition manufactured there is impressive. During World War II, the site produced over 4.3 billion rounds, or about 10% of small arms ammunition manufactured in the U.S. during the war (Thomson and Mayo 1991). During the Korean and Vietnam wars, the site produced over 11.4 billion rounds of ammunition (Hess 1984).

In addition to producing ammunition, TCAAP hosted other military-related activities. The site manufactured certain munitions-related components and stored materials for other government agencies. Private companies, including Honeywell and Minnesota Mining and Manufacturing Co. (3M Company), leased portions of the property for defense research and development.

Proximity to abundant water was critical for siting munitions plants (Hess 1984). Water fulfilled multiple needs, including serving as a source of energy, providing a raw material used in manufacturing process, and providing a receptacle for waste. During World War II, 502,966 gallons of raw water were pumped per million rounds of manufactured ammunition (Williams 1945—NARA Chicago), underlying the importance of access to an abundant local water supply.

While in operation, TCAAP generated military and industrial waste, containing explosives, heavy metals, pesticides, polychlorinated biphenyls (PCBs), and volatile organic compounds. Most of this was disposed of on-site, using dumping, burial, and open incineration. These practices led to extensive soil, sediment, and groundwater contamination. In 1983, the EPA designated TCAAP as a Superfund site—the largest in the state. But the site’s dumping of wastes off-site, into Lake Superior some 150 miles away, has received little attention.

Before 1972, when the Federal Water Pollution Control Act tightened regulations on water quality, Sect. 13 of the Rivers and Harbors Act of 1899, governed American dumping into navigable waters. Under this act, the Corps was allowed to dump any type of military waste into rivers, streams, and lakes, requiring prior approval only from the Secretary of the Army, rather than from state or local officials (Adamkus 1977—KAML&A).

Nevertheless, the choice of Lake Superior as a dump site for TAACP appears puzzling on the surface. One hundred and fifty miles is a substantial distance to transport toxic waste for dumping, contradicting the logic of proximity between military armaments’ production and their disposal in inland waters (Charrière 2019). Why did the Army decide that dumping these items in a distant lake was a satisfactory and economically efficient way to eliminate them?

As Tarr (1996) argues, tensions develop between the production of waste and attempts to dispose of that waste with minimal costs and maximum efficiency. Lake Superior, in Tarr’s phrase, became the “ultimate sink” for the military. For each of the two dumping campaigns, we argue below, the military argued that low costs were a key reason for choosing this method. In addition to this, for the dumping of barrels, there is the issue of the large accumulation of scrap material and the delay in acquiring equipment capable of recovering salvage of raw material. Moreover, the choice of Lake Superior was also motivated by the fact that the U.S. Army Engineering District St. Paul possessed the equipment and skills necessary because this type of service (immersion disposal) had already been carried out for other Department of Defense agencies (Walker 1959). As for the dumping of incendiary bullets, the military reasoned that because such waste could be risky for human safety, hiding them in a very deep lake might lessen those risks.

## Site A: Barrels dumping

In the winter of 1968, while trawling with his fishing vessel *Hiawatha*, Stanley Sivertson netted six barrels about 150 feet deep, in a site 0.75 miles from shore, only 1.5 miles east of the intake for the Duluth City water pumping system. Sivertson and his crew inspected the barrels onboard, and then dumped them back into Lake Superior in deep water. To understand the significance of Sivertson’s discovery, we must first review the operations conducted by Honeywell 150 miles away at the TCAAP.

Founded in 1885 as a company specialized in heating systems, Honeywell became a global technology leader after World War I. With the outbreak of World War II, the U.S. military awarded Honeywell defense contracts regarding engineering and manufacturing projects. Beginning in the 1950s, Honeywell leased buildings within the TCAAP facility to manufacture fuzes and ammunition for the Army (ARMCOM 1977).

By the late 1950s, scrap material from Honeywell’s production of anti-personnel grenades and rocket assemblies started to accumulate at Building 502 on the Twin Cities site (ARMCOM 1977). With the assistance of the Corps, Honeywell attempted different methods of disposal, including burning, explosion, and grounding. Eventually, while applying one of its core missions (explosive ordnance disposal), the Chicago Ordnance District chose freshwater dumping of material in 55-gallon steel drums called barrels as the most economical and secure method (Dean 1959). Once sufficient barrels had accumulated at the TAACP, they “were then transported by semi-truck under guard to Duluth or Superior, loaded on the [Corps’] barge and then [shipped] to a point selected by the tow Captain for dumping” (Heren 1977).

According to tugboat logs, seven barrel-dumping events occurred between October 1959 and September 1962 (USACE 1994: Appendices C, D and G). The exact locations of these dump sites are unknown, but Corps records initially specified that the minimum water depth was 100 feet and minimum distance from shore was 3 miles. In 1962, the Corps changed the required minimum depth to 300 feet (USACE 1994: Appendix G). During this three-year period, Honeywell contractors dumped over 1,400 barrels containing over 830,000 pounds of classified grenade scrap into Lake Superior.

What was in these barrels? Based on testimony of people who had firsthand knowledge or had participated in the disposal action, the contents of the barrels consisted largely of die-cast aluminum and steel scrap from the production in Building 502 (Heren 1977). In addition, no more than 6 barrels, loaded in the Honeywell Hopkins Plant located west in Minneapolis, contained “fiberglass tape impregnated with lithium chloride, potassium chloride, barium chromate, calcium chromate, and zirconium […] from a thermal battery used on a time fuze” (ARMCOM 1977: 2).

In 1959, the Army classified the production details of the antipersonnel grenades that Honeywell produced, to prevent enemy theft of design and manufacturing concepts. The by-products of this production were also considered classified material (USACE 1991: 1). Keeping new military technologies secret confers important advantage over enemies. For that reason, the disposal of the barrels was marked by secrecy: the shipment was closely guarded until its sinking (USACE 1994: Appendix G; ARMCOM 1977: Appendix). Not until 1974 was this information declassified (Quale 1959). As Colaresi (2014) argues, the value of secrecy decreases over time, and even more when new military technology has been revealed in the battlefield.

### Radioactive concerns

In the 1970s, three separate actors claimed that radioactive waste was hidden in the barrels. Rumors of radioactivity stirred immediate controversy that refused to be quelled by military reassurances. As the next section describes, military responses to media concerns about barrels dumped in Lake Superior reinforces Jacob Hamblin’s findings about marine disposal of nuclear waste (Hamblin 2002, 2006, 2008, 2014). Hamblin shows that military and governmental authorities once considered disposing of radioactive materials at sea as a feasible solution, giving relatively little thought to the potential risks posed to marine environments. Over time, concerns arose about the economic costs and potential threats to human health through marine food chains, but few concerns were expressed about effects on broader ecosystem.

In 1975, the League of Women Voters of Sault Ste Marie (MI) was the first actor to make public accusations about nuclear waste (Robinson et al. 1975—MTUA). The league reported that barrels dumped in Lake Superior were filled with atomic waste to Philip E. Ruppe, a Michigan representative, who sought clarifications from the Corps (Ruppe 1975; Noah 1975 MTUA).

In May 1976, during a handover briefing of the *Hiawatha*, Sivertson (the former captain) described his peculiar catches of six barrels in Lake Superior to Dan Rau, the new captain of the vessel (Rau 1976—Rau personal records). As vice president of the Save Lake Superior Association (SLSA), Rau was already sensitive to environmental matters. He contacted Corps and Army officers, as well as Minnesota Policy Control Agency’s employees, to obtain more information about those underwater barrels. He also contacted Twin Cities reporters.

Later that summer, Danforth Anderson, a retired Corps of Engineers tugboat captain, claimed that a military officer helping with the shipment told him that the barrels contained atomic waste from a plant outside St. Paul (*The Evening News*, August 27, 1976). Similarly, his wife Gertrude reported hearing rumor of containers filled with radioactive waste while present at the dock during one of the dumps (Braatz 1976).

In September 1976, a friend of the Anderson couple named Marilyn Burton spoke at a town meeting in Marquette Michigan, claiming as well that the barrels contained radioactive waste (*The Evening News*, August 27, 1976; Milliken 1977—Burton personal records). Burton’s claim prompted a series of exchanges between the Michigan Department of Natural Resources, the MPCA, and the U.S. Energy and Research Development Agency (ERDA). That same year, the ERDA quickly announced that no evidence of radioactive waste existed in their records (Liverman 1976—Burton personal records). But many locals remained unconvinced by this claim.

Local environmental advocates organized a flurry of regional news coverage, as well as the attention of political representatives and multiple governmental agencies, such as the Department of Defense (DoD), the EPA, and the MPCA. These local pressures led to three main periods of investigation: local investigations in the 1970s; federal investigations led by the Defense Environmental Restoration Program (DERP) for Formerly Used Defense Sites (FUDS) in the 1990s; and finally tribal investigations funded by Native American Lands Environmental Mitigation Program (NALEMP) in the 2000s.

The first study, conducted by the Corps with the help of the EPA in fall and winter of 1976, mapped the locations of 20 barrels (out of the 1437 dumped). No barrels were pulled up, and no contents were tested. Instead, the consultants tested the water quality surrounding the located barrels and stated that it was “good” with no indications of radioactivity (Environmental Research Laboratory-Duluth 1976). Local citizens were unsatisfied with those results.

The U.S. Army Armament and Material Readiness Command tried to quell radioactive rumors by locating more dump sites and determining barrel contents. The Command ordered the U.S. Army Environmental Hygiene Agency to conduct two assessments. The first focused on 6 barrels containing potential toxicants such as lithium chloride, barium, calcium chromate, and calcium chloride; the second focused on the 1440 barrels’ main components: steel and aluminum. The report from the U.S. Army Environmental Hygiene Agency concluded that the environmental hazards were negligible, except for chromium (low environmental hazard). The agency noted that its staff had been unsuccessful in their efforts to locate and recover most barrels, and so its report stated that it could not guarantee that there would be “no effect on the environment” (Piercy 1977). Nevertheless, the Army stated that the contents of the barrels did not have any significant toxic impact, chemical, physical or radiological, on the water and biological life in Lake Superior. As a result, they recommended discontinuing further research on the barrels (ARMCOM 1977).

Multiple stakeholders, including state agencies, environmental organizations, and political representatives, disagreed with this recommendation. For example, the MPCA requested on December 6, 1977, that the U.S. Army Armament Materiel Readiness Command continue its search for the barrels and retrieve them from each dump site for further analysis (Gardebring 1977—Burton personal records). On March 21, 1978, the Command held a meeting with the Corps, other Army representatives, the MPCA, the State of Michigan and Wisconsin, and environmental groups. At that meeting, the Army stated that “any further search for the barrels would be futile” (Moore 1978—Burton personal records). The *St Paul Pioneer Press* reported that, in the absence of new evidence, the Army would not resume the search for the barrels (March 22, 1978).

Investigations resumed following the Superfund Amendment and Reauthorization Act (SARA) of 1986. This act required that the Department of Defense (DoD), in consultation with the EPA, undertake environmental restoration programs at Formerly Used Defense Sites (FUDS). The EPA, the MPCA, and the DoD agreed to classify the Lake Superior Classified Barrel Disposal as a FUDS, one of 7060 such sites to be inspected for environmental damage (Patin 1990—USACE, Omaha District).

Radioactive concerns—never completely quelled by military assurances—burst into the media once again in 1990, after a Geiger counter registered radiation. On October 15, 1990, a Geiger counter inside the submarine of Captain Harold Maynard began to click, registering minute levels of radiation close to a barrel at Site A.^5^ The next day, Corps staff went with Captain Maynard to recheck the general area, but their Geiger counters found no indication of radioactivity. The Corps cast doubts on Maynard’s readings, calling his instrument “the unconfirmed Geiger counter” (Gardner 1991: 5). Maynard disagreed, arguing that that the Corps had not allowed him to scan the exact area where he had detected radiation the day before (Hazard Control 1990: Attachment E). Maynard accused the Corps of a cover up, while Mike Stich, another contractor present, argued as well that the Corps was downplaying the incident (Stich 1991—KAML&A). The local television station carried a special report on the Geiger counter readings, heightening interest.^6^

Between October 10 and November 26, 1990, the St. Paul District Army Corps of Engineers supervised the first phase of the FUDS investigation. Their contractors surveyed 24 square miles of the lake bottom, but located only one barrel dump site and removed only two barrels for inspection (Hazard Control 1990). Besides a significant amount of corrosion, the barrels contained cardboard boxes of unit packed metal timing and safety devices for BLU-3 and BLU-4 anti-personnel grenades, as well as M55 detonators (USACE 1991). The National Air and Radiation Environmental Laboratory did underwater testing in the vicinity of the barrels for radiation and found that none of the 24 scanned drums posed any radiological health or safety risks (NAREL 1990).

Based on findings from just one site, the Corps concluded that “the barrels observed on the lake bottom did not appear to be releasing material to the environment and the situation did not warrant a declaration of an emergency” (USACE 1991: 12). The MPCA and environmental groups disagreed, calling for the continuation of the project until all seven sites were identified and thoroughly surveyed. The DoD agreed to conduct more investigations (DERP-FUDS 1991—USACE, Omaha District), and in 1993, a sonar survey located 415 targets within 3 dump sites. In June 1994, 7 barrels were pulled up and their contents tested. Four contained the same materials as the barrel recovered in 1990, while the other 3 contained a mixture of aluminum, steel, lead, trash, and slag consisting of incinerated material from grenade assemblies (Oceaneering Technologies 1994a, 1994b). Water and slag from the barrels showed traces of fifteen chemicals, with PCBs, lead, cadmium, benzene, and barium all detected in levels higher than Minnesota water drinking standards (USACE 1994).

**Fig. 3:**
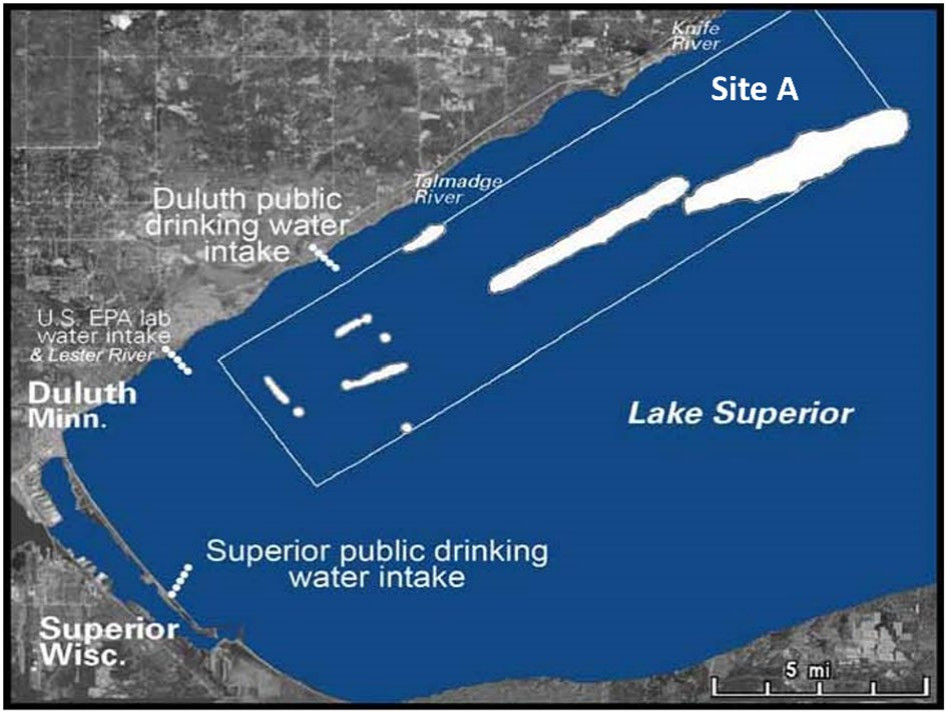
Barrels Dumping Areas (Site A) and Water Intakes. Credit: Bonnie Urfer, Nukewatch (2013)

At a meeting between the Corps, the MPCA, and the TCAAP, the Corps argued that the barrels’ location (over a mile away from water intakes—see Fig. 3) would dilute the chemicals to acceptable levels for drinking (MPCA, COE, TCAAP 1994—USACE, Omaha District). The MPCA agreed with the decision to suspend the search for the barrels. The state and federal agencies issued joint press release stating that the barrels did not pose an immediate threat to human health or the environment (*Duluth News-Tribune*, December 22, 1994a).

Local actors, however, remained unsatisfied, particularly concerned about claims that radioactivity existed, and evidence had been suppressed. The mayor of Superior, WI (located about seven miles from the first known dump sites) insisted that investigations continue. Congressman Oberstar (MN) contacted the EPA and requested a new radiological survey. In 1995, the National Air and Radiation Environmental conducted the requested survey on 24 barrels at three different dumpsites and reported that no radiation was detected (NAREL 1995).

These findings led the DoD in 1994 to label the Lake Superior Classified Scrap Disposal Site as a “No Further Action” site under the Formerly Used Defense Sites program. A decade later, the site was officially declared ineligible for the Defense Environmental Restoration Program—Formerly Used Defense Sites (Martin 2006—USACE, Omaha District).

### Tribal investigations

After the investigation surrounding the barrels in the 1990s was made public, a new stakeholder, the Red Cliff Band of Lake Superior Chippewa, became involved in this saga.

The Red Cliff Band is one of 154 bands of Anishinaabeg along Lake Superior who retained ceded territory rights in portions of the basin. Through a series of treaties signed with the United States, such as the Treaty at Prairie du Chien in 1835, Treaty at St. Peters in 1837, Miners Treaty in 1842, and La Pointe Treaty in 1854, Indigenous nations retained key rights on ceded territories, including the rights to hunt, fish, and harvest. However, the states largely ignored these rights until 1971, when in the case *People v. Jondreau*, the judge reaffirmed Lake Superior Chippewa rights outlined in the 1842 Treaty (Supreme Court of Michigan 1971).

The Red Cliff band became involved in the barrels controversy through two channels, one at the community level and the other at the federal level. First, a “few extremely active Red Cliff tribal environmental activists, including Walter Bresette, Jeanne Buffalo-Reyes and Judy Pratt Shelly, became engrossed in this issue and warned the tribe about it” (*Indian Country Today*, August 20, 2012). Second, studies show that Native Americans and their lands are disproportionately exposed to hazards posed by the U.S. military’s explosive and toxic munitions (Hooks and Smith 2004; LaDuke and Cruz 2013). In this regard, a broader theoretical framework applies to this situation, “slow violence”, which is defined by Nixon as “a violence that occurs gradually and out of sight, a violence of delayed destruction that is dispersed across time and space, an attritional violence that is typically not viewed as violence at all” (2011: 2). To address tribal concerns, since 1993 Congress has required the DoD to devote funds to mitigate environmental impacts on tribal lands. Accordingly, the DoD created in 1996 the Native American Lands Environmental Mitigation Program to address environmental impacts on tribal lands from former DoD activities and facilities.

The Red Cliff Band’s reservation lands and ceded territories have been “identified as having possible environmental impacts resulting from DoD activities” (Painter and Stacy 1997). The Army had approved the disposal of the barrels within the ceded territory, where recent court decisions have recognized that the Anishinaabeg tribes have authority over the management and oversight of Lake Superior natural resources, as stated in the 1842 Miners Treaty.

The tribes around the Great Lakes were concerned that the barrels may have contaminated the waters and may have harm fishing areas. Commercial and subsistence fishing have been part of Ojibwe lifeways since time immemorial. The Anishinaabeg view Lake Superior as sacred and considers its waters “as a Traditional Cultural Property in which ceremonies, spiritual, fishing and related cultural activities centered upon” (RCB 2006: 18).

As stewards of water, including Lake Superior, the Red Cliff Band decided to conduct new investigations, such as sediment sampling. Sediments have the capacity to absorb and accumulate dissolved nutrients and chemicals, as well as store pollutants for extended periods of time, releasing them gradually over decades. Therefore, sampling sediments can be an effective means of detecting anthropogenic water contamination (Ayrault et al. 2021; Parrinello and Kondolf 2021). It is striking that in all the years of testing conducted by the Army and state authorities, no sediment sampling had been done.

The Native American Lands Environmental Mitigation Program identified the Lake Superior barrels to be eligible for the program, given the continuing uncertainty about the risks they posed to the lake’s water quality. This program allows tribes, as sovereign nations, to work with the U.S. government, specifically the DoD and the Corps, in a government-to-government relationship. The DoD and the Red Cliff Band signed a cooperative agreement in September 2004, with the intent “to fully characterize the type and extent of DoD waste that impacts the economy, natural resources, and culture of the Chippewa Ceded Territory and to completely remove the DoD waste” (RCB 2006: 9).

Between 2008 and 2017, the Red Cliff Band led several investigations organized cooperatively with multiple agencies. For example, in 2012, contractors recovered 25 underwater barrels (EMR 2014). This investigation detected still-active explosives, identified as active ejection charge composed of M5 propellant. No previous study had identified such items. Because these were explosive devices, the Department of Transportation, required that the tribe obtain additional permits (RCB 2014) which further delayed the project schedule and sparked more questions about the barrels.

EMR, a contractor for the Red Cliff Band, conducted remedial investigations in 2013 and 2014, including an ecological screening and a human risk assessment; these represent the most comprehensive investigations of the barrels to date. The findings indicated that the waters and sediment within the investigation area contained potential contaminants, such as polycyclic aromatic hydrocarbons (PAH) and volatile organic compounds (VOC), at or above laboratory detection limits. “However, interpretation of the barrels’ contributions to such contamination is obscured by the fact that many of these contaminants are also present in background samples” (EMR 2014: 85). EMR concluded that “due to their remote location and the current levels of contaminants present, a significant human health and ecological risk is not currently identified” (EMR 2014: 85). Nevertheless, EMR recommended further evaluation of drinking water and human consumption of fish, which is of particular concern for the Ojibwe as wild fish is an important source of nutrition for them, and tribal members may be at greater risk, given high levels of fish consumption (Hoover 2013).

The Minnesota Health Department had argued in 2008 that “the risks of detrimental exposures to people from these barrels are unquantifiable, but low”, while suggesting that aquatic species faced potentially larger risks than people (MDH 2008: 14). This reasoning assumed that human risks could be isolated from risks to the fish, a logic that rests on the premise the human and environmental health can be separated (Beck et al. 2018; Maser and Strehse 2021; Sanderson et al. 2017). Yet, for the Ojibwe, human health emerges in relationship with environmental health, rather than in isolation (Langston 2010). The premise that fish might be harmed by the barrels through the process of bioaccumulation, while the people who consume those fish would remain unharmed, is problematic for the Anishinaabeg.

The Red Cliff Band continued their investigations for another 4 years, concluding in 2017 with a sonar search that identified over a thousand-barrel targets. This study provided the most comprehensive map of the barrels’ location (1021 out of 1437). The study recommended further investigation to provide sufficient data for effective decision-making (Ridolfi 2017). But despite the Red Cliff Band’s desire to continue the investigations, the DoD suspended the program in 2018.

## Site B: Bullets dumping: 1945

The practice of dumping waste, including military waste, into bodies of water has been prevalent for centuries. However, the historical context surrounding this practice is specific to each individual site, raising questions about under what conditions military authorities deem that underwater dumping is appropriate, and how the military handles civilian concerns. By contrasting the barrels dumping (Site A) with the bullets dumping (Site B), we have the opportunity to identify both the similarities and differences in military waste management.

Site B (Fig. 2) contains incendiary bullets, also called hot bullets, dumped in the 1940s from the TCAAP plant 150 miles away. After World War II, the commanding officer at TCAAP defended the underwater dumping operations as a means of enhancing safety and reducing costs. Burial, burning on land, or storing the bullets, the commander argued, would all present more significant hazards than underwater dumping (*Duluth News-Tribune*, October 3, 1945a). In addition, the commander noted, underwater disposal would save the taxpayers approximately $20,000 (*Pioneer Press*, September 29, 1945).

Press reports released to papers near the dumping site provided readers only vague information about dumping locations. These locations were not only vague; they were also contradictory. For example, one article stated that the material must be disposed in 85 feet of water (*Duluth News-Tribune*, October 3, 1945a), while others stated that it was in 600 feet of water (*Minneapolis Morning Tribune*, September 29, 1945). Although the press reported that 600 feet of water would “avoid the danger of water pollution,” (*Duluth Herald*, September 28, 1945), the article did not clarify who determined that 600 feet limit would be safe, and for what organisms. Nor did it clarify what type of pollution was involved, and how those materials might affect aquatic ecosystems or drinking water quality. With the publication of these articles, the Federal Cartridge Company recognized that favorable illustrated newspaper displays presented these dumping operations in a positive light by emphasizing the security and economic aspects (FCC 1946—NARA Chicago). In 1945, some citizens did express skepticism about military disposal into Lake Superior—but this skepticism was based on a concern about costs, not on environmental or health concerns. For example, an anonymous Duluth resident wrote to the local paper that they expected better use of war bond funds and suggested instead converting the waste “into peacetime uses at a profit to the government” (*Duluth News-Tribune*, September 29, 1945c). Another resident asked why underwater disposal was chosen instead of surplus salvage (*Duluth News-Tribune,* October 3, 1945b). Indeed, at the end of World War II, the disposal of surplus war supplies was a contentious topic, with debates surrounding the goals to be pursued and the type of administration in charge of such programs (Dobney 1974). One major concern for Congress was that the disposal of surplus war supplies should not unduly interfere with the operations of private businesses in the postwar period. The proposed pattern of disposal generally included seven categories, ranging from transfer within the government to the destruction of unneeded materials, including the sale or lease to agencies of foreign governments (Huddle 1943). A large portion of the surplus war materials would have little value except as scrap. In this context, building 513 at TCAAP was transformed into a “reclamation center” for salvaging metal scrap from unusable small arms ammunition until 1951. During this period of eight years, approximately 550 million rounds of ammunition were processed, and significant contributions were made to salvage technology (Hess 1984).

Congressional Representative William A. Pittenger (MN) demanded fuller explanation from the War Department about the economic rationale for dumping rather than re-use. In a letter to the Chief of Ordnance, he asked “Why is it [the dumped material] not made available for civilian use and just how much consideration was given to the taxpayer in connection with this program?” (*Sunday Pioneer Press,* October 7, 1945).

Until 1993, few citizens or politicians pursued questions about the bullet dumping program, until news of the dumped bullets re-emerged during barrel investigations conducted by the DoD at Site A. The remote operation vehicle^7^ searching for barrels close to Duluth instead discovered an underwater military landfill containing a box of bullets (Oceaneering Technological 1994a). This discovery sparked a brief period of citizen concern, and the MPCA conducted a short investigation, soon concluding that no risks were present (*St. Paul Pioneer Press*, November 28, 1993; *Duluth News-Tribune*, May 29, 1994d). Public interest quickly faded.

These two dump sites into Lake Superior present fascinating comparisons. Both dump sites originated from the same military production facility 150 miles away. Both dumping operations involved a similar amount of material, and many uncertainties remain at both sites. Yet Site A sparked decades of intense controversy, scientific investigations, and skepticism about military assurances of safety. In contrast, Site B raised little initial citizen concern, and when the state and Army claimed the materials were safe, the public appeared to accept these claims. Why did these differences arise?

We had hoped to answer these questions by scrutinizing the details of the MPCA’s state report on Site B’s safety. But much to our surprise, no copies of these reports appear to have survived. We know the report once existed, because a letter from the Army written in 1994 advised the MPCA to write a report outlining about Site B, so that “appropriate project recommendations can be submitted to higher authority for review and approval” (MPCA, COE, TCAAP 1994—USACE, Omaha District). In 1995 the Commissioner of the MPCA wrote to the Mayor of Superior that the MPCA was “continuing a background search into the allegation of the dumping of ammunition in 1945” (Williams 1995). Finally, the U.S. General Accounting Office mentioned that “the state pollution agency and Corps of Engineers had not yet decided whether an investigation by the Army of the 50-caliber bullet disposal was necessary at the time of our review” (GAO 1997: 18), suggesting the report was still being completed.

However, the MPCA and the state archives were unable to locate for us the 1990s report from the MPCA that they used to justify a decision that the site posed no risks. Nor could they locate for us any of the documents and evidence they had gathered to substantiate their claim that the materials were safe. We could find no evidence that a thorough investigation had ever been conducted on the bullets.

Could the materials dumped at Site B be harmful to the aquatic ecosystem, to fish, or to the people and other animals that consumed the fish? This question is unanswerable. We do know that underwater military munitions “present challenges due to […] potential ecological and human health impacts resulting from the release of munitions constituents to the aquatic environment” (Lotufo et al. 2021: 1). We also know that in 2013, the *Duluth News Tribune* reported that the dumped bullets might have contained white phosphorus, a highly toxic substance (March 2, 2013). Yet the puzzling absence of public concern and investigation has persisted. Local governments, citizens, and NGOs have never raised serious concerns about the dumping at Site B, even though controversy continues about Site A, just a few miles away. The resources of tribal governments and grassroots associations are not unlimited. Therefore, they must prioritize their environmental struggles. The environmental concerns affecting Lake Superior are numerous, and have required significant support from these groups, such as addressing the adverse effects of mining industries (Langston 2017). As a result, other concerns deemed less important have been set aside, such as the bullet disposal. Perhaps this situation provides us with another example of how secrecy can lead to rumors and speculation, as its operations always leave traces. On the other hand, transparency can help alleviate suspicion surrounding government and military activities.

## Other dumping sites within the Great Lakes

Was other military waste dumped into Lake Superior? During our investigation, we found suggestions that the Army may have organized additional dumping operations into Lake Superior. First, a record dated September 29, 1959, reveals that the Corps from St. Paul District had prior experience in underwater disposal of material for other DoD agencies (Walker 1959). Second, in 1976 a local paper reported that “we were repeatedly told that at that time [1959–1961] it was a common practice to dispose of classified materials in Lake Superior.” The article added that “we have found no evidence of any other occurrence” (*The Chequagemon Sun*, August 19, 1976). In the third bit of evidence, a former Corps supervisor “reported that there were at least 16 disposal sites where the Army dumped everything imaginable, including a lot of ammunition” (*St. Paul Pioneer Press*, November 28, 1993). Our search of archival records and agency reports, however, did not uncover additional information about the other 14 alleged sites.

Historians investigating ocean dumping of chemical weapons argue that because of military secrecy, the details may never be fully known (Greenberg et al. 2016). The barriers encountered in mobilizing knowledge regarding underwater munitions are numerous and cannot be solely concealed under the seal of military secrecy. As Souchen (2021c) explains, shaping archival records depends on a set of factors, such as national security concerns, access and information laws that apply to both governments, private military contractors, and historians. Other factors directly related to underwater sites, such as dumping of materials outside agreed official dumping areas and the movement of materials due to geophysical movement, lead to a loss of knowledge related to these munition deposits (Beddington and Kinloch 2005). The same is likely true, we argue, for Great Lakes dumps. Both marine and freshwater disposal of military waste reflects the willingness to hide these materials beneath the water, where the public is unlikely to venture. One appeal of military water dumping has long been its ability to put knowledge about the waste “out of reach” (Müller and Stradling 2019). Yet the idea that dumping military waste puts it out of reach is merely an illusion, because toxic water contamination easily moves through aquatic food chains into human bodies.

Within the other Great Lakes, freshwater military waste dumping sites also existed. Yet, as Table 1 shows, the quantity of materials dumped into the two Lake Superior case we have discussed (Sites A and B) accounted for a significant proportion of known dumping sites.

**Table 1:** Military Dumping Within the Great Lakes

Country	Lake	Quantity	Dumping years
Canada	Lake Huron	Over 2.2 million pounds of ammunition	1945
United States	Lake Ontario	Unknown amount and types of materials	During World War II
United States	Lake Superior	Over 1 million pounds of bullets	1945
United States	Lake Superior	Over 830,000 pounds of classified grenade scrap	1959–1962

Sources: For the Lake Huron, see *The Globe and Mail*, November 21, 1945. For the Lake Ontario, see Kennedy and Kappel 2000

## Media analysis

Sometimes underwater dumping does stay hidden from public concern, as Site B suggests. But other times, underwater dumps seem to raise even greater concerns, as Site A shows. To explore the reasons for this difference, we examine print media coverage to explore the roles of secrecy and rumors in generating public concern and action. Scholars have shown that news media play key roles in constructing public knowledge (Heinz 2005; Lahtinen and Vuorisalo 2004), so analysis of media patterns can help historians understand contrasts between secrecy and public disclosure of environmental risks.

In the 1940s, our analysis shows, when the earliest news articles about Site B were published, those articles came not from an independent media investigation, but rather from a press operation organized by Army itself, during the dumping operation itself. War time press censorship existed, and the media generally agreed with the need for such censorship to protect national interests (Gottschalk 1983). At the end of World War II, good relationships existed between the TCAAP and the local press, who published a series of favorable feature articles on plant and its post-war plans (FCC 1946—NARA Chicago). The first articles about the bullet dumps appeared on September 28, 1945 (*Duluth News-Tribune*; *Duluth Herald)* and the last ones on October 25, 1945 (*Green Bay Press-Gazette*; *The Journal Times*; *The Oshkosh Northwestern*).

By the 1950s, the military’s media strategies had shifted. Press censorship of military matters continued, but media outlets were becoming less willing to accept military assurances that the national interest required such censorship. The military found that its cordial relations with media outlets was eroding (Porch 2002). Neither the Army nor Honeywell organized media coverage while dumping the barrels at Site A at the end of the 1950s. We found no official justification explaining this lack of publicity and apparent secrecy. Site A’s dumping operation occurred as the Cold War intensified, and the Army chose to make the barrel contents classified (which they had not done with the bullets at Site B a decade earlier). Classifying the materials made it more difficult for potential Soviet spies to steal the details of antipersonnel grenades—but classifying these materials added a layer of secrecy that contributed to public concern about radioactive wastes.

By the mid-1970s, anti-war protests and opposition to U.S. military actions had furthered transformed relations between the military and the media. Local and national media became more interested in publishing materials critical of military activities. We see this reflected in a growing number of media reports about the barrel dumping in the 1970s. In 1976, the first local article about the barrels in Site B was published by *The Chequagemon Sun* (August 19, 1976). Over the next four decades, hundreds of media articles were published; the most recent is dated (*Cook County News Herald*, February 28, 2022). Review of their contents reveals three main findings. 

First, we observe that military attempted to construct a thorough communications and public relations plan to control the information disseminated. Müller’s 2016 analysis of the dumping of chemical weapons stocks in the ocean is particularly interesting in this context. Originally conducted by the DoD in absolute secrecy, Operation CHASE (for “Cut Holes and Sink ‘Em”) was meant never to be made public. However, in the summer of 1969, Congressman McCarthy made the dumping program public. This political bombshell led to numerous Congressional hearings, multiple science commissions, and an extensive media campaign. While the DoD tried to restore its credibility, its action proved unsuccessful due to significant gaps in knowledge. We suspect that in order to avoid a repeat of the CHASE disaster, the army and authorities decided to conduct their investigation into Site B’s with complete transparency. During the investigation in the 1970s, reporters and observers were invited to accompany the Corps aboard a tugboat to observe attempts to find and identify the barrels (ARMCOM 1977). Two decades later, when concerns about the barrels re-surfaced, the Corps established a temporary public affairs center in Duluth to answer all informational requests and provide daily press releases (USACE 1991: Appendix E). From the Corps’ position, both media plans worked well, leading them to praise the fair coverage of the search by the press. During the last search, the Red Cliff Band, as lead investigator, adopted a different approach. No media was allowed to be on any research vessel and no daily press conference was organized. Deprived of their right of access, some journalists expressed their dissatisfaction about the Red Cliff Band’s public relations plan on several occasions (*Superior Telegram*, August 9, 2012; *Reader Weekly*, March 13, 2014). Due to the magnitude, significance, and innovation of this project, the Red Cliff Band chose to manage their communication by allowing them to share their findings at their own pace. To accomplish this, they established a blog called “Red Cliff Band of Lake Superior Chippewa—Lake Superior Barrels Project”^8^ and held many public information sessions to share updates on the project, release press statements, and provide other relevant information pertaining to the barrels.

Second, we observe that the media has presented a wide range of perspectives from the stakeholders^9^ involved in the barrels story, including their support, concerns, and complaints regarding the management of this issue. The newspaper articles have reported on the positions of some stakeholders, such as the Army and Save Lake Superior Association, since the 1970s. However, only recently have they covered the actions and concerns of the Red Cliff Band. The tribe’s position on the issue was only reported for the first time in 2005 (*Duluth News-Tribune*, April 29, 2005). This coincides with a grant given to them from the Native American Lands Environmental Mitigation Program. This situation highlights a broader trend: the lack of coverage of Indigenous environmental justice issues in mainstream media. This finding supports the argument of Moore and Lanthorn (2017) that Indigenous people face significant challenges in getting adequate media coverage when dealing with environmental issues.

Third, our review shows that only a few articles published in the mid-1990s have mentioned the story of the bullets at Site B and always as a side note to the barrels at Site A. In analyzing the hundreds of articles published about the barrels at Site A, we find that the millions of U.S. dollars spent on their investigation since the mid-1970s, and the host of agency reports claiming few toxic risks, have not lessened public concern about the barrels. Rather, the opposite may be true: the more the agencies insist the risks are few, the less the public trusts their claims of safety. We suspect that the initial secrecy offered by classification of barrel contents may have backfired, intensifying distrust. The fact that the 1945 bullets dumping at Site B was not classified, and that the Army organized press releases at the time in an appearance of openness, may have contributed to the attenuation of public concern.

## Persistence of rumors regarding the radioactivity of the barrels

Since the barrels resurfaced in the mid-1970s, rumors of radioactivity have persisted despite official denials. The Army used two methods to deny the presence of radioactivity: scientific evidence and witness statements. The military conducted water tests in 1976 and found no evidence of radioactivity (Environmental Research Laboratory-Duluth 1976). Former workers at TCAAP testified that neither specific protection, nor special safety instruction was provided during the loading of the barrels (Rothman 1976; Ruby 1977). A veteran truck driver contractor for Honeywell transported the barrels from the TCAAP to Duluth recounted the same story in the press: “No precautions, such as wearing protective clothing or monitoring the loads for radioactivity, were even taken” (*St. Paul Pioneer Press*, November 11, 1976). However, sceptics pointed out that the lack of special precautions didn’t prove that hazards had necessarily been absent. For example, when dumping radioactive waste from nuclear projects into the ocean, “American sailors were told that this was harmless and given no special training to eliminate unfamiliar by-products of the nuclear age then emerging” (Reno 2019: 9). Rumors have persisted even in the face of official denial. Similarly, in the chasm of Jardel, located in France, rumors about the presence of mustard gas in shells persist despite official investigations stating otherwise (Charrière 2019).

Three specific events have contributed to the lack of trust some citizens and environmental groups have regarding military narratives regarding the barrels at Site A. First, armed guards were present when the barrels were transported from the TCAAP to Duluth. Citizens ask: Why would the Army have armed guards if the barrels were only filled with harmless scrap materials? However, armed guards were also present when barrels were transported to incineration furnaces (*Duluth News-Tribune*, November 16, 1976a). During the Cold War years, classified military materials were often accompanied by extra guards, even when radioactive materials were absent.

Second, distrust has also persisted because radioactive particles are imperceptible without the intervention of scientific devices, such as Geiger counters. And Geiger counters play an important role in the spread of the rumor, as described earlier. While the Army instruments have showed no radioactivity, other Geiger counters were claimed to have shown evidence of radioactivity. The general public has been reluctant to believe Army statements, given the widespread media attention to hidden nuclear exposure at other military sites such as Hanford (Brown 2013).

Finally, we suspect that the rumor of radioactivity has persisted because both Honeywell and 3M Company had been licensed to use radioactive material at the TAACP. The U.S. Atomic Energy Commission gave a license for uranium to Honeywell on February 5, 1969, roughly 7 years after the last dumping occurred (AEC 1969—KAML&A). 3M Company was issued a license to use radioactive from May 9, 1961, through May 2, 1967 (NRC 2000). The discovery of a secret landfill containing radioactive waste outside of Kerrick,^10^ Minnesota, used by 3M Company between 1966 and 1969, has led some citizens to suspect that the company may have added radioactive waste to the barrels dumped in Lake Superior (*Duluth News-Tribune*, November 20, 1994b). Additionally, the discovery of depleted uranium and other radioactive elements in the sewers of the building 502 (ITC 1990) where the barrels originated has reinforced suspicions about their radioactivity. Yet no hard evidence links 3M Company or Honeywell’s radioactive materials with the dumped barrels.

Another rumor regarding a “purplish goo” oozing from a burst barrel only added to the public distrust (*Duluth News-Tribune*, April 21, 1990). No certainty about the origin of this substance emerged after several investigations (MDH 2008; Dempsey 2008), allowing speculation to flourish.

The Corps and state agencies continue to argue that the barrels are harmless and have no toxicity or radioactive materials. Some local citizens and environmental NGOs remain distrustful, believing that the barrels contain toxic and hazardous materials. According to them, no hard evidence exists to prove that during the seven dumping operations the same innocuous materials were filled inside the barrels. Up to now, the recovered barrels tend to serve their point of view, because each time new barrels were recovered, new contents were discovered, strengthening the lack of trust towards the Army and governmental agencies. Retrieving all the barrels and studying all their contents might lessen this trust, but the logistics would make that task impossible. In the 1990s, Ron Swenson, a former MPCA employee in charge of the Lake Superior barrels investigation, said: “There may be no way to discount the possibility some of the barrels contained radioactive waste short of exhuming all of them from the lake’s bottom. […] So, the mystery of radioactive waste is still out there. Did they do it and, if so, where are the barrels?” (*Duluth News-Tribune*, November 20, 1994b).

## Management complexities

The reality of underwater military waste and associated environmental damage is often difficult to sense, and even more difficult to witness. Searching for military waste lying in deep waters has its own technological difficulties, leading to weakened management effectiveness. National boundaries further complicate management. Even though Lake Superior is managed by Canada as well as the United States, we found no direct involvement from Canadian federal authorities or the International Joint Commission.

The lack of records regarding the barrels is widely recognized. Back in 1976, a Pentagon spokesperson said he was unable to locate records of the scrap dumped into Lake Superior (*Duluth News-Tribune*, August 21, 1976b). In 1977, a civil engineer from the Corps signaled that the historical records available “are limited due to required record destruction and the sinking of one tugboat and the burning of another” (Hager 1977). In the 1990s, a Brigadier general of the Army gave another justification: “As public waters, the disposal area has no ownership or disposal records” (Patin 1990—USACE, Omaha District).

Although military guidelines specified that the barrels had to be disposed of at depths of at least 100 feet, some barrels were found at 50-foot depths—intensifying public concern (ARMCOM 1977). Contractors might have ignored their initial dumping guidelines, starting to dump the barrels along the way to the designated areas, a common practice known as *en-route* dumping (Böttcher et al. 2011, 2015). As Souchen argues (2021a, 2021c, 2021d), the military frequently relies on private contractors to execute operations involving the disposal of munitions into water. Their involvement often complicates the military’s overall control of the process. Alternatively, the barrels might have been moved by currents or trawling (ARMCOM 1977). Even with specialized and costly equipment, surveying underwater barrels remains challenging when their locations may be shifting.

Each period of investigation ended with the same conclusion: the barrels are not harmful; no further investigation and no cleanup are needed. While some actors have always disagreed with this conclusion, others have come to accept these conclusions. For example, as described earlier, the MPCA initially rejected the Corps’ conclusion that the barrels were safe (MPCA 1994—KAML&A). But by the end of 1994, the state of Minnesota concurred with the military (Williams 1995).

Two conflicting positions currently prevail. The leading actors of the first position include federal and state agencies, as well as some local citizens and journalists. They argue that the barrels contain only scrap materials, which can be labeled as debris and do not require remedial action. As a result, the *status quo*—leaving untouched the barrels on the lakebed—can be maintained. Another group of actors, including the Save Lake Superior Association and the Red Cliff Band of Lake Superior Chippewa, take a different position. Save Lake Superior Association now requests the recovery of barrels located near Duluth’s water drinking intake, because of potential risks to human health (SLSA 2016).

The Red Cliff Band is dissatisfied with the *status quo* decision as well, but their concerns extend beyond direct human health concerns, to fisheries and cultural values. They wish to test a representative sample of barrels from across all sites (not just those sites close to human drinking water intakes). Since 2018, the Corps have been working on a memo describing the contents of the barrels, but the Red Cliff Band has not yet received any updates (*Cook County News Herald*, February 28, 2022). Additionally, the tribe’s military grants for these investigations have ended in 2017, and their applicants for further funding have been denied.

## Military dumping into Lake Superior: An incomplete picture

Besides the environmental hazards that underwater military waste might cause, dumping operations demonstrates how waste management decisions can backfire. In the late 1950s, underwater dumping was justified by cost efficiency. But after decades of strife, over $4.5 million has been spent on search-and-recovery efforts, with little resolution. The secrecy that accompanied the initial barrel dumping was intended to protect classified information, but it too has backfired. Unknowns, such as the contents and location of the dumped barrels, continue to multiply, sparking continued debate and leaving the burden of responsibility for future generations.

In addition to the lack of proximity, the choice of Lake Superior as the “ultimate sink” (Tarr 1996) to permanently dispose of military waste from a military industrial site (TCAAP) is not surprising in itself. However, the study of these two examples (Site A and Site B) demonstrates that the examination of the environmental impact of past U.S. military activities must be considered in an integrated manner (from war preparation to post-war, including armed conflicts) and take into account all environmental compartments (soil, water, and air).

Local, grassroots pressure was critical in forcing the military and state authorities to investigate the barrels—even though no cleanup operation has yet been implemented. It is crucial to note that without the mobilization of local citizen associations and Indigenous peoples, no effort to assess the risks associated with these barrels would have been undertaken.

The full story behind the disposal of bullets and barrels may never be known. The gray areas surrounding both events are numerous and finding reliable sources of information from the disposal period is challenging. The acknowledgement of two sites of military waste in Lake Superior may represent only the tip of the iceberg. Numerous sites of military production, testing, and dumping happened in the Great Lakes region during the twentieth century, even though few have been closely examined by historians or environmental agencies. Examining their environmental legacies should be encouraged to protect the largest freshwater system in the world.

Globally, maintaining the *status quo* is a common approach for underwater military munitions sites, including those in oceans, seas, and lakes. Authorities typically argue that underwater munitions should remain undisturbed because they do not pose an acute threat to either human beings or the environment. But this perspective ignores the human cultural connections to water. More comprehensive cleanups would consider cultural, moral, historical, and spiritual perspectives on water as well as health and environmental concerns. Simply transferring the problem to future generations is not a sustainable solution.

## Footnotes

1 A complete view of all American Formerly Used Defense Sites can be viewed on online: https://ags03.sec.usace.army.mil/portal/apps/webappviewer/index.html?id=5a541ac5c0064c01a685a72f16854fbf. Accessed on 10 January 2023.

2 https://fudsportal.usace.army.mil/. Accessed 22 Dec 2022.

3 We met these actors twice: three through videoconference, two over phone, and one in-person. In addition to videoconferences’ recording and personal notes, the interviewees agreed to share reports and personal records with us.

4 Government-owned, contractor-operator (GOCO) facilities are partially or wholly owned federal industrial plants built with government funding and operated by selected contractors. Each U.S. Department of Defense branches controlled their respective GOCO facilities. However, only a few GOCO facilities remain active today. Most of them, whether closed or opened, have legacy contamination that still needs to be remediated (Robert and Cobb 2017).

5 More specifically: “a continuous series of clicks from the instrument and a reading of approximately 20–40 counts per minute on the 0.1 × scale, which would correspond to an exposure rate of 0.1–0.2 mR/h” (NAREL 1990: 1).

6 Available online: https://www.youtube.com/watch?v=uUk9jcmEv_Y. Accessed 9 Jan 2023.

7 A remotely operated vehicle (ROV) is a robot submarine that can seek out targets hundreds of feet under water and relay pictures to vessels on the surface.

8 Available online: https://lakesuperiorbarrels.wordpress.com. Accessed 1st Nov 2022.

9 The main stakeholders involved in this issue includes the Corps, the DoD, the MPCA, the EPA, environmental groups (e.g., Save Lake Superior Association and Nukewatch), federal and local representatives, local citizens, the Red Cliff Band, etc*.*

10 More specifically, the landfill included two main disposal sites: one containing 154 barrels of volatile organic compounds (VOCs); and one of 652 barrels of low-level radioactive waste containing Uranium and Thorium (MPCA 1997: 107).
